# Surveillance and characterisation of influenza viruses among patients with influenza-like illness in Bali, Indonesia, July 2010–June 2014

**DOI:** 10.1186/s12879-019-3842-5

**Published:** 2019-03-07

**Authors:** Wiku Adisasmito, Sri Budayanti, Dewi Nur Aisyah, Richard Coker, Ayu Rai Andayani, Gavin J. D. Smith, James W. Rudge

**Affiliations:** 1 Faculty of Public Health, Universitas Indonesia, Depok, West Java 16424 Indonesia; 2 Udayana University, Denpasar, Bali Indonesia; 3 Bali Provincial Health Office, Denpasar, Bali Indonesia; 4 Communicable Diseases Policy Research Group, Department of Global Health and Development, London School of Hygiene and Tropical Medicine, London, UK; 5 Faculty of Public Health, Mahidol University, Bangkok, Thailand; 6 Duke-NUS Medical School, 8 College Road, Singapore, Singapore

**Keywords:** Influenza, Active surveillance, Virus, Indonesia, ILI

## Abstract

**Background:**

Although Indonesia has high fatality rate of human A/H5N1 cases, epidemiological and clinical data on influenza virus circulation among humans has been limited. Within Indonesia, Bali province is of interest due to high population densities of humans, pigs and poultry. This study aims to characterize and compare the epidemiological and clinical patterns of influenza viruses in humans through surveillance among patients with influenza-like illness (ILI) in Bali, Indonesia.

**Methods:**

ILI patients were recruited at 21 sentinel health facilities across all nine regencies in Bali, from July 2010 to June 2014. PCR-based assays were used for detection and subtyping of influenza viruses. Demographic, behavioural and clinical data were tested for associations with influenza using chi-squared tests and logistic regression.

**Results:**

Of 2077 ILI patients, 291 (14.0%) tested positive for influenza A, 152 (7.3%) for influenza B, and 16 (0.77%) for both influenza A and B. Of the influenza A isolates, the majority 61.2% were A/H3N2, followed by A/H1N1-pdm09 (80; 26.1%). Two A/H5N1 were identified. Influenza positive rates were significantly higher during wet season months (28.3%), compared with the dry season (13.8%; χ^2^ = 61.1; df = 1; *p* < 0.0001). Clinical predictors for infection varied by virus type, with measured fever (≥38 °C) more strongly associated with influenza B (AOR: 1.62; 95% CI: 1.10, 2.39).

**Conclusion:**

Influenza circulates year-round among humans in Bali with higher activity during the wet season. High contact rates with poultry and pigs, along with influenza virus detection that could not be subtyped through conventional assays, highlight the need for molecular studies to characterize epidemiological and evolutionary dynamics of influenza in this setting.

**Electronic supplementary material:**

The online version of this article (10.1186/s12879-019-3842-5) contains supplementary material, which is available to authorized users.

## Background

The Asia-Pacific region plays a key role in the emergence of novel influenza viruses, as well as global transmission of seasonal influenza viruses [1, 2]. Indonesia, the largest nation in Southeast Asia, is of particular importance for influenza surveillance, as it is endemic for highly pathogenic avian influenza A/H5N1 in birds [3–5], and has the second highest cumulative number of reported human cases H5N1 worldwide. Since 2003 until April 2016, there were 199 confirmed human H5N1 cases recorded in Indonesia, with a case fatality rate among the highest globally at 83% [6]. Furthermore, H5N1 has also crossed the species barrier into pigs in Indonesia [7–9]. This is of concern, as pigs may act as a “mixing vessel” for avian, swine and human influenza viruses and serve as a source for the emergence of novel reassortant viruses, since the trachea of pigs contains receptors for both avian and human influenza viruses [10]. Such concerns were elevated with the emergence of a new influenza A virus (A/H1N1-pdm09) in 2009 which caused our most recent pandemic, with fears that this strain could genetically re-assort with other influenza viruses, such as H5N1, to generate a new pandemic strain of higher pathogenicity.

Within Indonesia, the island province of Bali is arguably of special interest for influenza surveillance and research, due to a variety of cultural, ecological and socio-economic factors that converge here to make it a potential hotspot for the emergence and spread of influenza viruses [11, 12]. For example, the predominance of Hinduism in Bali, in contrast to the Muslim-majority populations in many other parts of Indonesia, means that pork production and consumption are relatively high in Bali. The pig population in 2013 was estimated at almost 1 million, the second highest among all Indonesian provinces [13], with pigs often raised in rural smallholders systems which also keep poultry. In addition, with a geographic area of 5633 km^2^ and a human population of around 3.9 million, Bali has a high human population density, and is also a major tourist destination, hosting an estimated 9 million visitors in year [14]. Together, the high densities of, and close interactions between, human, pigs and poultry, along with the high mobility of the human population, provide fertile conditions for cross-species and human-to-human transmission, and make the island a potential “mixing bowl” for influenza viruses from a wide range of geographic regions.

Considering the convergence of these factors on Bali, along with the high incidence and rapid mutation rate of influenza viruses, continuous monitoring of circulating influenza strains is extremely important. This project aims to help rectify the lack of knowledge on influenza in Indonesia, by conducting four years of influenza surveillance among humans presenting with influenza-like illness at health facilities across Bali. This study was initiated following the emergence of influenza A/H1N1-pdm-09 in 2009 with specific objectives to identify the influenza strains circulating among humans in Bali, and describe their epidemiological, clinical and virological characteristics.

## Methods

### Study design

Four years of active surveillance of influenza viruses among patients presenting with influenza-like illness (ILI) was conducted in Bali province, Indonesia, from July 2010 until June 2014. Patients were recruited from the outpatient departments of 21 sentinel health facilities (10 government hospitals and 11 urban health centers) across all eight regencies and the provincial capital city, Denpasar (in Indonesia, regencies and cities are the second-level of administrative sub-division, between province and district). ILI was defined as measured fever of ≥38 °C at time of presentation, or self-reported recent history of fever (in the last 10 days), and at least one upper respiratory symptom. This is similar to the current WHO recommended ILI case definition (“an acute respiratory illness with a measured temperature of ≥ 38°C and cough, with onset within the past 10 days”) [15], but not identical, since the latter was published in 2011, after the surveillance period for our study had begun. In addition to measured fever, self-reported fever was included in our case definition, based on observations by local health authorities that patients with fever often self-administer analgesics such as acetaminophen (which can reduce fever) prior to seeking health care.

A systematic interval sampling approach [15] was applied to ensure samples were obtained across all regencies of the province throughout the year. Specifically, every month each sentinel site was requested to recruit at least the first two consenting patients presenting with ILI, in order to achieve our target of recruiting at least 2000 ILI patients in total over the study period. Monthly intervals were chosen for sampling quotas in order to give sufficient temporal resolution to identify patterns in virus circulation, whilst acknowledging that weekly sampling intervals would overburden health facilities with limited capacities, and also may not be feasible during periods of relatively low ILI incidence. There was some variation in actual monthly sampling rates between facilities, with some unable to meet their quota of two patients every month (for example due to low numbers of ILI presentations, limited capacities, and turnover of designated staff), while other facilities often exceeded their quota. Despite this variation, the sampling design enabled us to meet our overall goal of recruiting at least 2000 ILI patients throughout the study period, with samples obtained from every regency of Bali throughout the year.

### Questionnaire administration and specimen collection

Written informed consent was obtained for all participants included in the study. For every patient meeting the inclusion criteria, designated staff within the health facility asked for their willingness to be involved in the research. Afterwards, staff explained the purpose of this research as shown in information sheet. If they agreed, they were asked to sign the informed consent letter. For participants under 18 years old, written informed consent was obtained from their accompanying parent or guardian.

Upon recruitment of ILI cases, a questionnaire was administered by health staff to collect data on demographic variables, symptoms, any underlying medical conditions, and potential exposure-related risk factors (including recent contact patterns within humans and animals). Information on any treatments prescribed to the patient was also recorded. The questionnaire was developed following consultation with local health authorities and health workers and was pilot-tested at health facilities prior to implementation.

Nasopharyngeal swabs were obtained from enrolled ILI patients, placed in viral transport medium, and refrigerated at 4 °C until they could be transported to the laboratory at Udayana University in Denpasar. Samples were transported to the laboratory within 48 h after collection, where they aliquoted and stored at -70 °C until processing. The mean duration between sample collection and testing was 18 days.

### RNA extraction and qRT-PCR for influenza diagnosis and subtyping

At the virology laboratory in Udayana University, all collected swabs were subjected to a diagnostic assay to detect and differentiate between influenza A and B using reverse transcription-PCR (RT-PCR). RNA extraction was performed using QIAmp viral RNA mini kit (Qiagen). Influenza A positive samples were subjected to subtyping of the haemaglutanin gene using single reactions, following WHO recommended protocols [16]. Detailed procedures can be found in the Supporting Information.

### Data analysis

Data were double-entered and cleaned in an Excel database, and statistical analyses were performed using *R* version 3.3.2 (https://cran.r-project.org/). Descriptive analyses were performed to investigate the distribution of virus strains by age group, geographic area, and month. Laboratory-confirmed influenza among ILI cases was tested for associations with demographic, behavioral and clinical variables in univariable analyses based on Chi-squared tests, and multivariable analyses using logistic regression. In these analyses, four influenza diagnostic outcome variables were considered separately: laboratory confirmed infection with any influenza virus, and laboratory confirmed infection with each of the three most common influenza types/subtypes (A/H3N2, A/H1N1-pdm09, and B). Patient age group, sex, and any variables found to be associated with any of the influenza outcomes with *P* < 0.1 in univariable analyses were subsequently included as independent variables in the multivariable logistic regression models. Hospitalization was also examined as outcome variable to test for associations with age, sex, pre-existing respiratory conditions, and influenza diagnosis using a similar approach. Statistical significance was assessed based on *P* < 0.05.

## Results

### Study participant characteristics

Over the 4-year study period, 2077 patients with ILI symptoms were recruited in the study area. The mean age of patients was 17.0 years old (range 0.5–80 yrs), 55.6% were male, and three quarters (75.4%) were recruited at primary health centers, with the remainder recruited at hospitals. In 2010, 162 (7.8%) patients were sampled, followed by 641 (30.9%), 506 (24.4%), 520 (25.0%), and 248 (11.9%) in years 2011, 2012, 2013, and 2014 respectively. Based on location, a quarter (25.5%) of study participants were recruited at the sentinel sites in Denpasar, followed by Badung (16.7%), Tabanan (14.5%), Klungkung (12.2%), Buleleng (11.2%), Jembrana (9.3%), Bangli (4.1%), Karangasem (3.7%), and Gianyar (2.8%).

### Influenza diagnoses

**Fig. 1:**
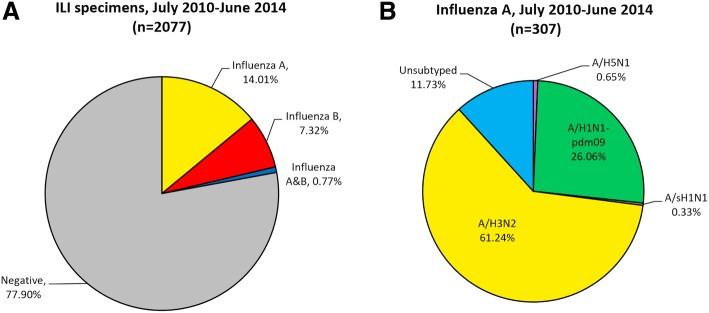
Diagnostic test results of 2077 Patients with ILI Symptoms (**a**), and subtype distribution of influenza A samples (**b**)

Of all 2077 specimens tested using real time-PCR assays, 291 (14.0%) tested positive for influenza A, 152 (7.3%) for influenza B, and sixteen (0.77%) tested positive for both influenza A and B (Fig. 1a). Of the 307 influenza A samples, 188 (61.2%) were subtyped as A/H3N2, 80 (26.1%) as A/H1N1-pdm09, and one (0.3%) as seasonal A/H1N1 (Fig. 1b). Thirty-six (11.7%) of the influenza A positive swabs tested negative in all subtyping assays, and were therefore of undetermined subtype. This may have been due to a low viral load in these samples, resulting in the positive targeting of the M gene, but negative detection of the HA gene in subtyping assays. No co-infections with multiple influenza A subtypes were detected. We also obtained samples from two suspected avian influenza A/H5N1 cases (a sister and brother aged 5 and 10 respectively; both fatal) in October 2011, which were confirmed as A/H5N1. It should be noted, however, that these A/H5N1 cases were not recruited through the systematic interval sampling of ILI cases presenting at our sentinel facilities. Rather, these two cases had been hospitalised at Sanglah Hospital with suspected avian influenza, and specimens were purposively sampled and sent to Udayana laboratory for testing.

### Spatial and temporal patterns

**Fig. 2:**
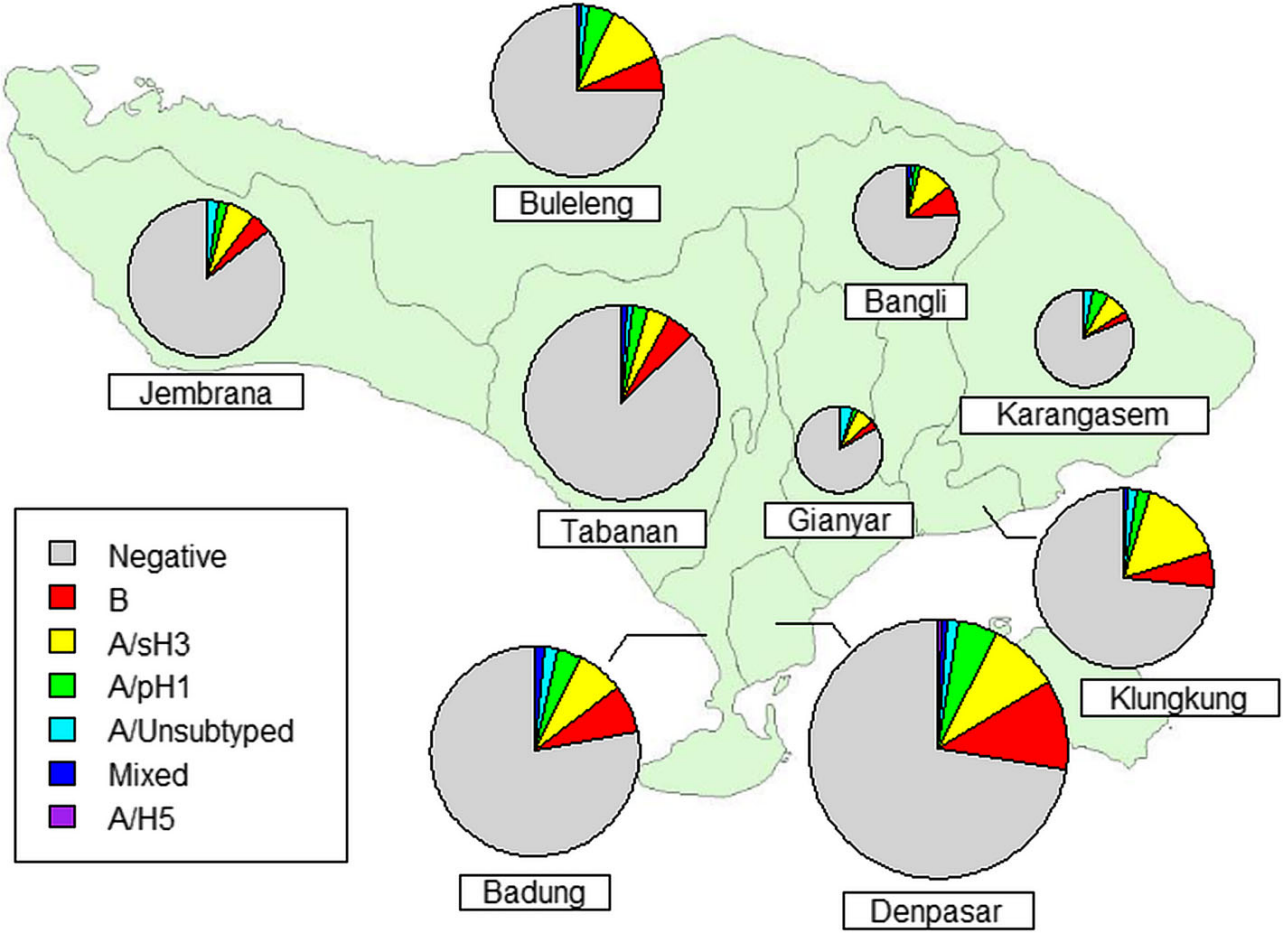
Spatial distribution of influenza viruses among patients presenting with influenza-like illness in Bali, Indonesia. The area of each pie chart is proportional to the number of ILI patients sampled. The map in this figure was generated on our own

Patterns of influenza virus distribution were broadly similar across geographic areas (Fig. 2). However, there was significant variation by regency in the proportion of ILI cases testing positive for influenza (χ^2^ = 37.7; df = 8; *p* < 0.001), which ranged from 12.6% (Tabanan) to 27.6% (Denpasar).

**Fig. 3:**
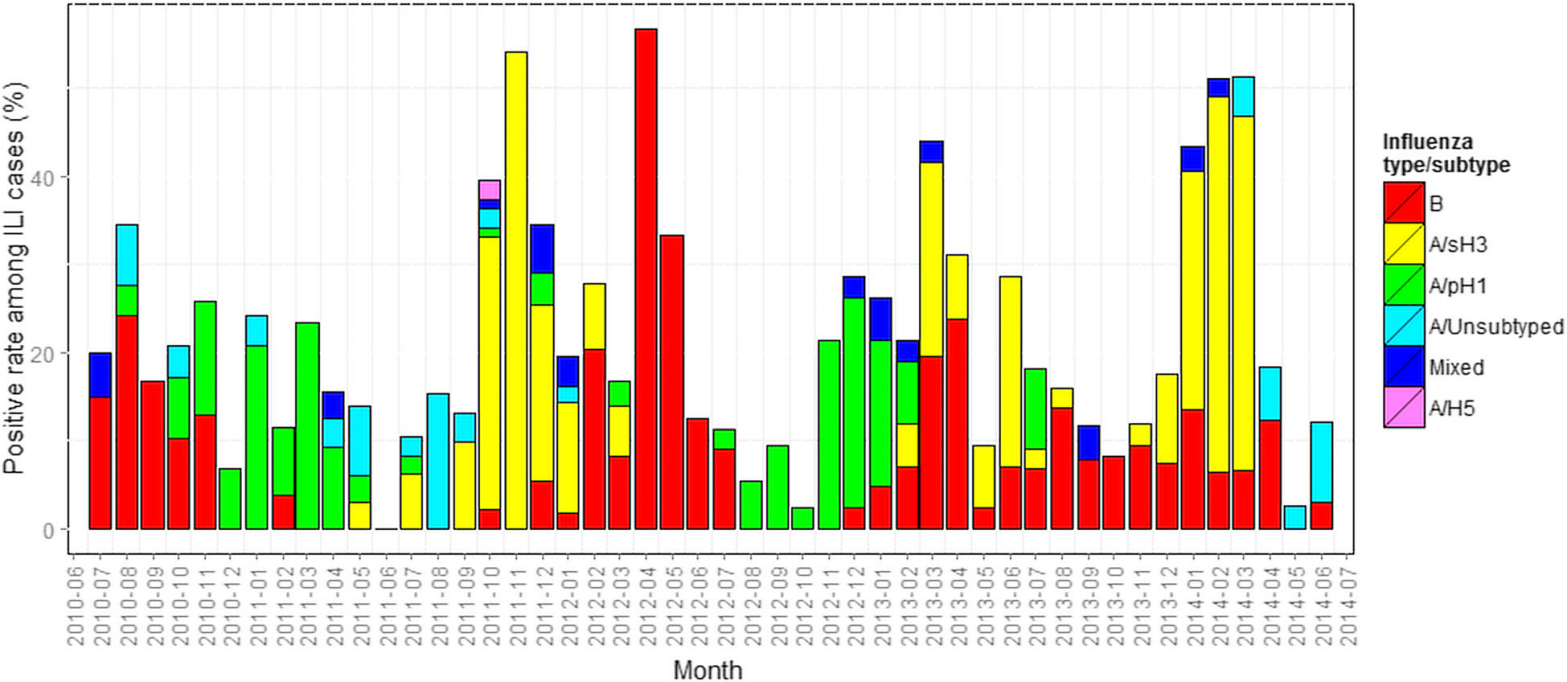
Temporal patterns of influenza virus detection among ILI patients in Bali between July 2010 and June 2014

Influenza viruses were detected in every month throughout the study period (Fig. 3), although influenza positive rates among ILI specimens were significantly higher during Bali’s wet season months (i.e., between October and April), compared with the dry season (May–September) (28.3% vs 13.8%; χ^2^ = 61.1; df = 1; *p* < 0.0001). The association with wet season was stronger for influenza A subtypes compared with influenza B (Fig. 3; Table S1 and Table 1).

**Table 1:** Multivariable logistic regression analysis of clinical variables associated with laboratory confirmed influenza among ILI patients in Bali

	Any Influenza (A or B)			Influenza A/H1N1-pdm09			Influenza A/H3N2			Influenza B		
	AOR	(95% CI)	*P*	AOR	(95% CI)	*P*	AOR	(95% CI)	*P*	AOR	(95% CI)	*P*
ILI symptom onset in wet season	**2.41**	**(1.90,3.04)**	**< 0.001**	**3.46**	**(1.93,6.23)**	**< 0.001**	**4.97**	**(3.24,7.61)**	**< 0.001**	1.37	(0.98,1.92)	0.07
Measured fever ≥38 °C	1.22	(0.95,1.57)	0.13	0.91	(0.53,1.57)	0.74	1.05	(0.73,1.52)	0.78	**1.62**	**(1.10,2.39)**	**0.02**
Measured fever ≥39 °C	1.29	(0.93,1.77)	0.12	1.79	(0.97,3.30)	0.06	1.53	(0.98,2.38)	0.06	0.59	(0.34,1.04)	0.07
Rhinorrhoea	1.24	(0.97,1.58)	0.09	0.78	(0.48,1.27)	0.33	**1.72**	**(1.17,2.52)**	**0.01**	1.01	(0.71,1.45)	0.95
Chest pain	1.43	(0.81,2.55)	0.22	0.70	(0.16,3.05)	0.64	**2.49**	**(1.23,5.01)**	**0.01**	0.70	(0.25,2.00)	0.51
Abdominal pain	0.81	(0.49,1.33)	0.41	1.30	(0.53,3.16)	0.57	**0.35**	**(0.12,0.98)**	**0.05**	1.10	(0.55,2.19)	0.79

AOR, adjusted Odds Ratio. (Values are adjusted for age, sex, and other clinical variables in the table)

CI, confidence interval

Data in bold are statistically significant

Patterns of differential temporal circulation of virus type/subtypes were evident, with each of the three main circulating influenza viruses showing two or three dominant phases throughout the study period. Specifically, influenza B was predominantly detected in Jul-Oct 2010, Feb-July 2012, and March, April & August 2013, influenza A/H1N1-pdm09 in Nov 2010-Apr 2011 and August 2012-Feb 2013, and influenza A/H3N2 in Jul 2011-Jan 2012, March and June 2013 (Fig. 3).

### Clinical presentation

**Fig. 4:**
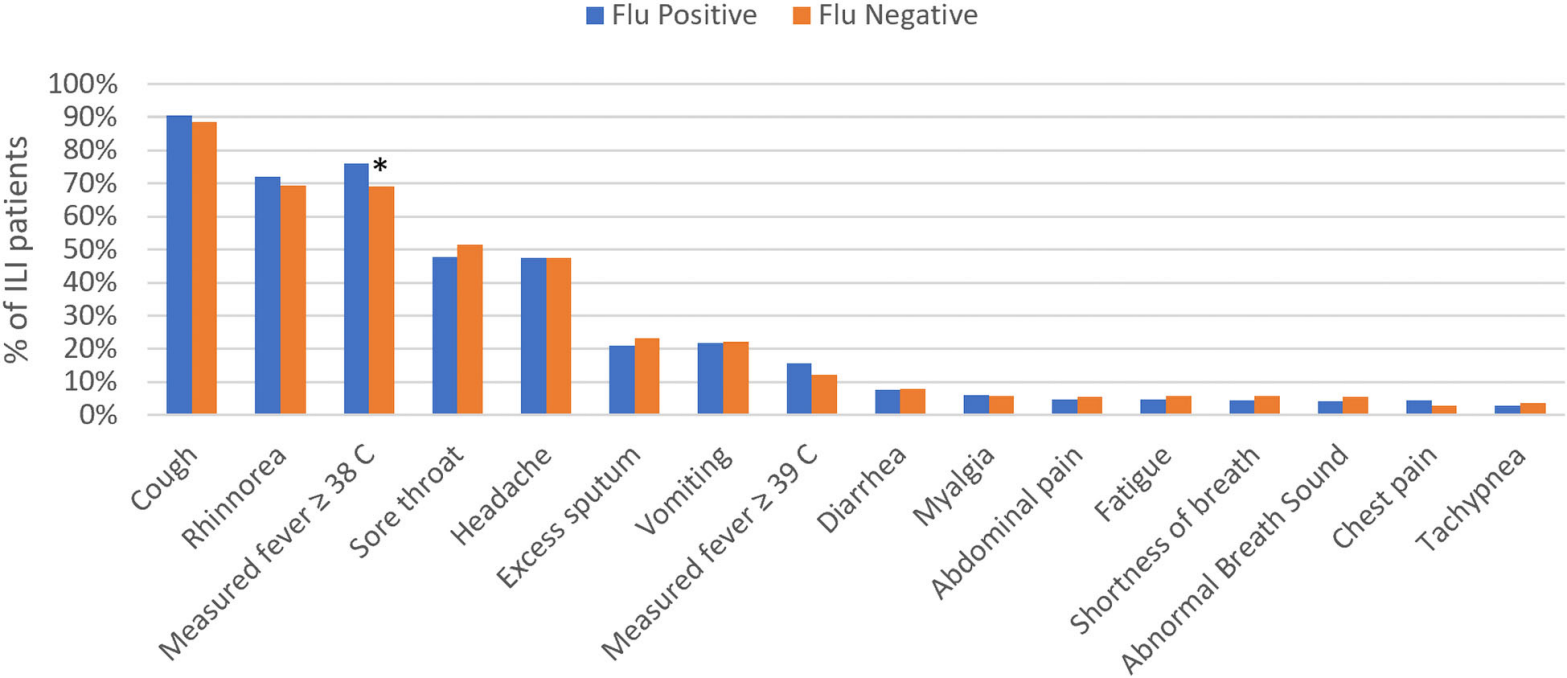
Prevalence of symptoms among ILI patients in Bali (*denotes significant association with influenza test positivity at P < *0.05)*

The prevalence of symptoms among ILI patients at time of recruitment are shown in Fig. 4, and univariable and multivariable analyses for associations with influenza test results are presented in Additional file 1: Table S1 and Table 1, respectively. Cough, rhinorrhea, and a measured fever (≥38 °C at time of presentation) were the most common symptoms, reported among 89.1, 70.0, and 69.0% of ILI patients, respectively. In univariate analysis, onset of ILI symptoms during the wet season was significantly associated with all influenza diagnostic outcomes. In addition, patients with measured fever ≥38 °C were significantly more likely to test positive for influenza (odds ratio [OR]: 1.41; 95% confidence interval [95% CI]: 1.1–1.8), and specifically influenza B (OR: 1.54; 95% CI: 1.05–2.26), while those with measured fever ≥39 °C were more likely to test positive specifically for A/H1N1-pdm09 (OR: 1.93; 95% CI: 1.09–3.42). Rhinorrhea (OR: 1.65; 95% CI: 1.15–2.37) and chest pain (OR: 2.19; 95% CI: 1.15–4.18) were significant clinical predictors of A/H3N2, while patients with abdominal pain (OR: 0.36; 95% CI: 0.13–0.98) were less likely to test positive for this virus (Additional file 1: Table S1). Multivariable regression models, incorporating all symptom variables that showed significant associations in univariate analysis, and adjusting for age and sex, found symptom onset in the wet season to be the strongest clinical predictor of laboratory confirmed influenza, and specifically influenza A subtypes (adjusted OR for A/H3N2: 4.97; 95% CI: 3.24–7.61). Measured fever was the strongest clinical predictor for influenza B (adjusted OR: 1.62; 95% CI: 1.10–2.39), but was no longer significant for other influenza outcomes in the multivariable analysis (Table 1).

There was no significant association between influenza positivity and the type of facility (hospital vs health centre) at which ILI patients were sampled (Additional file 1: Table S1). In terms of treatments, antibiotics and antiviral drugs were prescribed to 71.3 and 2.2% of ILI patients, respectively (noting that prescriptions were made based on clinical assessment by the health worker, before our influenza laboratory test results were available). The most commonly prescribed antiviral medication was oseltamivir, which accounted for 34 (73.9%) of the 46 reported antiviral prescriptions. ILI cases who tested positive for influenza A/H3N2 were significantly more likely to have been prescribed antivirals (OR: 2.34; 95% CI: 1.15–4.75) (Additional file 1: Table S1).

### Demographic and risk factor assessment

**Fig. 5:**
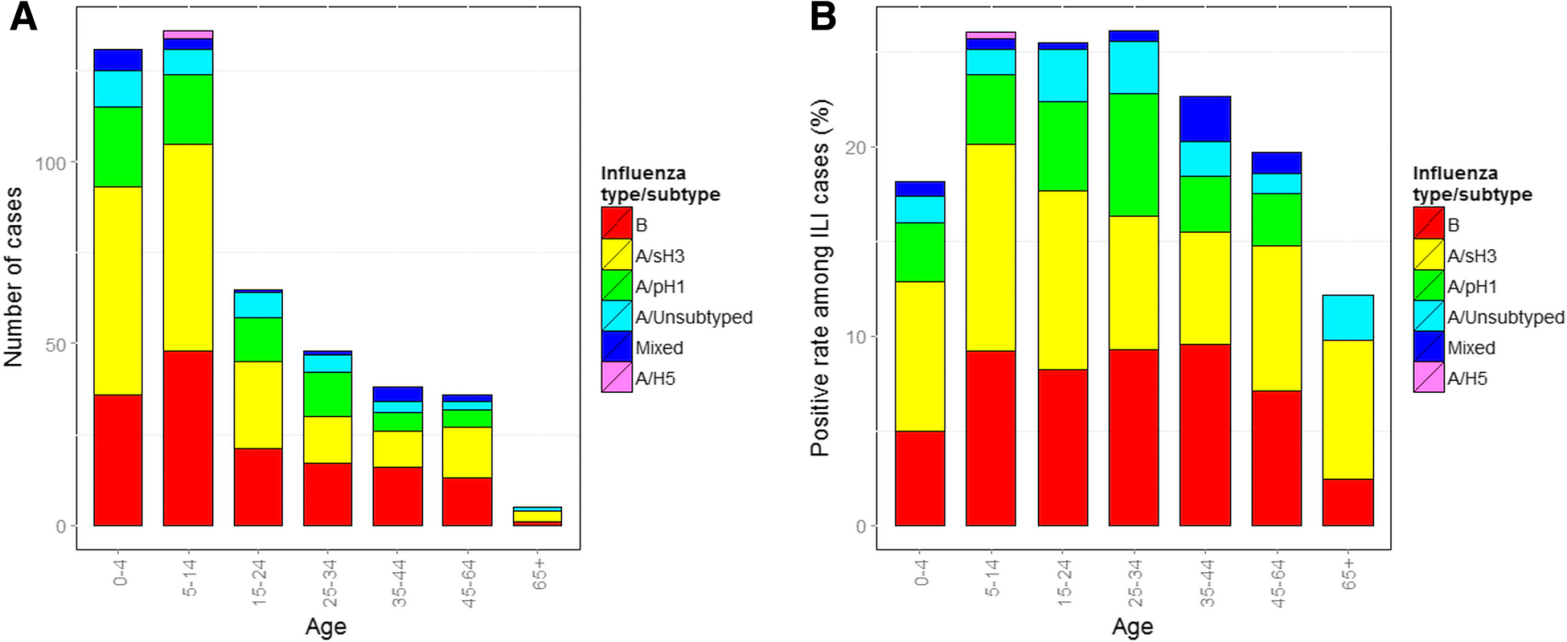
Number of influenza positive samples (**a**), and influenza positive rates (**b**) among ILI patients in Bali by age-group

Results of regression analyses of demographic and other risk factors for associations with influenza diagnosis are given Table 2. The highest number of influenza cases was observed in age group 5–14, followed by age 0–4, with 58.4% of all influenza positive samples originating from these age groups. This reflects the relatively high number of ILI patients recruited from these age-groups, rather than higher influenza positive rates among children with ILI (Fig. 5). Relatively few (39) patients aged ≥65 years were recruited, of which five (12.8%) tested positive for influenza (Fig. 5a). The proportion of ILI cases testing positive for any influenza virus differed significantly between age groups (χ^2^ = 17.6; df = 6; *p* = 0.007), and was highest in those aged between 5 and 14 years (Fig. 5b). No significant associations were found between influenza virus detection and sex, smoking, or having a pre-existing chronic respiratory condition (Table 2).

**Table 2:** Univariable and multivariable logistic regression analysis of demographic and behavioural variables for associations with laboratory confirmed influenza among ILI patients in Bali

	Univariable associations with influenza positivity			Multivariable associations with influenza positivity		
	Crude OR	(95% CI)	*P*	Adjusted OR	(95% CI)	*P*
Age group (y) (ref: 0–4)						
5–14	**1.59**	**(1.21,2.08)**	**0.007**	**1.48**	**(1.10,1.99)**	**0.04**
15–24	**1.54**	**(1.10,2.16)**		1.35	(0.94,1.96)	
25–34	**1.59**	**(1.09,2.32)**		1.47	(0.99,2.19)	
35–44	1.32	(0.88,1.98)		1.20	(0.78,1.83)	
45–64	1.10	(0.73,1.66)		0.84	(0.53,1.32)	
65+	0.63	(0.24,1.62)		0.64	(0.24,1.67)	
Male sex	1.00	(0.81,1.23)	1	0.97	(0.78,1.20)	0.76
Pre-existing chronic respiratory condition	0.71	(0.33,1.52)	0.37	–	–	
Smoker	0.64	(0.25,1.68)	0.37	–	–	
No. of face to face contacts per day (ref: 0–4 persons)						
10–49	**1.39**	**(1.10,1.77)**	**0.002**	1.19	(0.93,1.54)	0.18
50+	**1.77**	**(1.25,2.51)**		1.41	(0.97,2.06)	
Sick contact with ILI*	1.08	(0.77,1.50)	0.67	–	–	
Recent contact with poultry^T^	**1.33**	**(1.04,1.70)**	**0.02**	1.26	(0.89,1.78)	0.20
Recent contacts with pigs^T^	**1.40**	**(1.05,1.88)**	**0.02**	1.15	(0.86,1.54)	0.35

OR, Odds Ratio

CI, confidence interval

*Contact with another person with ILI within 14 days prior to symptom onset;

^T^Physical contact with live, sick or dead animals within 14 days prior to symptom onset

Data in bold are statistically significant

**Fig. 6:**
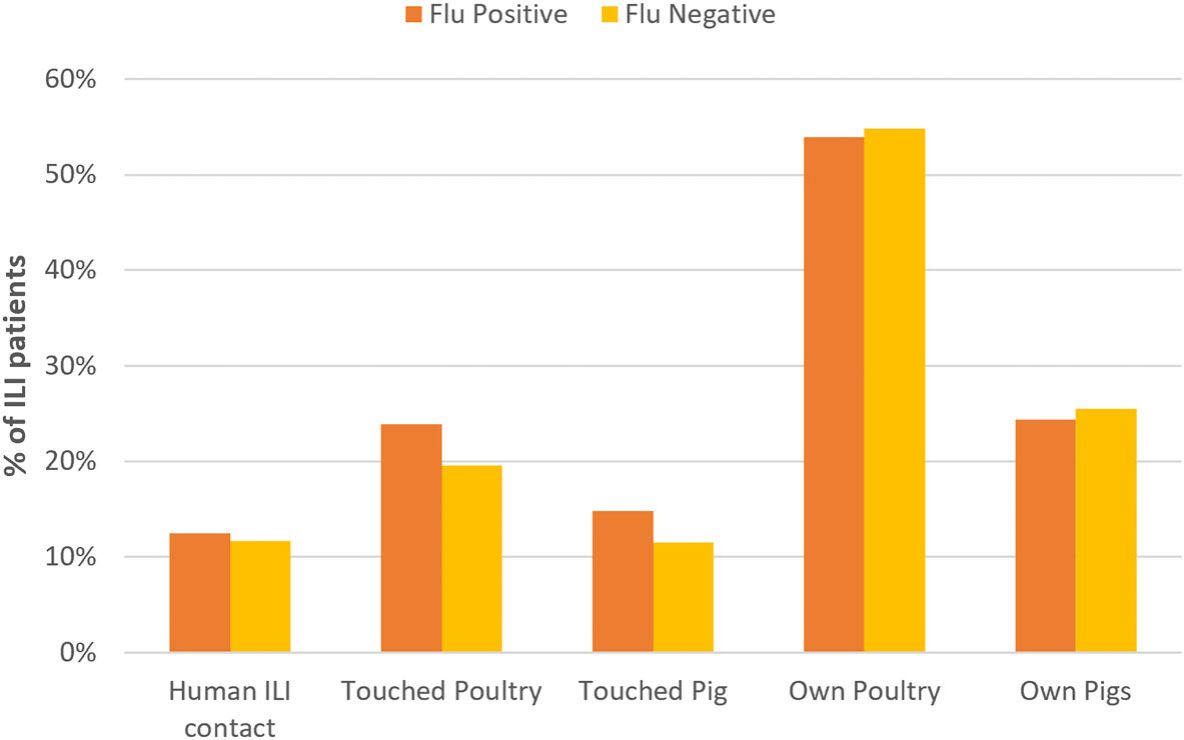
Frequency of recent contact with humans with ILI, and contact and ownership of pigs and poultry, among patients with ILI in Bali

In terms of behavioural risk factors, ILI cases reporting a higher estimated number of face-to-face human contacts per day tended to be more likely to test positive for influenza, although this was not significant when adjusting for other variables (Table 2). Approximately 12% of ILI cases reported contact with another person with ILI symptoms within 14 days prior to their own symptom onset; this was not significantly associated influenza positivity. Frequency of variables related to poultry and pig exposure were high among the study population, with 54 and 25% of ILI patients living in households that own poultry and pigs, respectively, and 20 and 12% of patients reporting recent physical contact (within 14 days prior to symptom onset) with poultry and pigs, respectively (Fig. 6). Furthermore, recent contact with poultry and pigs were both significantly associated with influenza infection in univariate analysis (OR: 1.33; 95% CI: 1.04–1.70 and OR: 1.40; 95% CI: 1.05–1.88 respectively), but not when adjusted for other variables in multivariable analysis (Table 2).

### Hospitalizations

A total of 145 hospitalizations were reported among 1981 subjects for whom data on this were available, giving an overall ILI case hospitalization rate of 7.3%. Hospitalization as an outcome variable was significantly associated with age-group, pre-existing chronic condition, and influenza test results in both univariate and multivariable analyses (Table 3). ILI cases testing positive for unsubtyped influenza A were more than three times more likely to have been hospitalized compared with influenza-negative ILI cases, when adjusting for age, sex, and having a pre-existing chronic condition (adjusted OR: 3.40; 95% CI: 1.28–9.30). Meanwhile, influenza B cases were less likely to be hospitalized than influenza-negative ILI cases, although this was less significant in multivariable analysis. Being in the youngest age group (0–4 yrs), and having a pre-existing chronic respiratory condition were also significant risk factors for hospitalization (Table 3).

**Table 3:** Univariable and multivariable logistic regression analyses of factors associated with hospitalization among ILI patients in Bali

	Univariable association with hospitalization			Multivariable association with hospitalization		
	Crude OR	95% CI	*P*	Adjusted OR	95% CI	*P*
Age group (y) (ref: 0–4)						
5–14	**0.27**	**(0.17,0.44)**	**< 0.0001**	**0.25**	**(0.15,0.41)**	**< 0.0001**
15–24	**0.09**	**(0.03,0.26)**		**0.09**	**(0.03,0.24)**	
25–34	**0.06**	**(0.02,0.25)**		**0.06**	**(0.01,0.24)**	
35–44	**0.10**	**(0.03,0.33)**		**0.10**	**(0.03,0.34)**	
45–64	**0.16**	**(0.06,0.40)**		**0.16**	**(0.06,0.39)**	
65+	0.30	(0.07,1.26)		0.25	(0.06,1.11)	
Male sex	1.41	(1.00,2.00)	0.05	1.30	(0.90,1.88)	0.17
Pre-existing chronic respiratory condition	**4.34**	**(2.20,8.57)**	**< 0.0001**	**5.55**	**(2.61,11.81)**	**< 0.0001**
Influenza diagnosis (ref: Negative)						
B	**0.35**	**(0.13,0.96)**	**< 0.0001**	0.45	(0.16,1.25)	**0.0003**
A/H3N2	0.61	(0.29,1.27)		0.68	(0.32,1.43)	
A/H1N1-pdm09	1.28	(0.58,2.86)		1.71	(0.73,3.99)	
A/Unsubtyped	**2.50**	**(1.02,6.15)**		**3.4**	**(1.28,9.02)**	

OR, Odds Ratio. (Adjusted OR values are based on multivariable logistic regression adjusting for all other variables in the table);

CI, confidence interval

Data in bold are statistically significant

## Discussion

Over 4 years of influenza surveillance among ILI patients in Bali between 2010 and 2014, this study found 22.1% of patients with ILI were related with laboratory confirmed influenza. This is generally within the range (~ 10–22%) found across studies in other countries in Southeast Asia [17–22], and highly consistent with national surveillance data for Indonesia reported by the Ministry of Health (MoH), in which influenza-positive rates among ILI patients ranged between 20 and 23% across years 2011–2014 [23–25]. Our Balinese data were also consistent with available nationwide data in terms of the relative frequencies at which different influenza virus types and subtypes were detected, with our data showing comparable positive rates for influenza A (14.0%) and B (7.3%) to those reported by MoH in 2014 (12.6 and 7.4%), and similar dominance of influenza A/H3N2 during this period [25].

A strength of our study is the use of 21 sentinel health facilities across all eight regencies and the provincial capital city (Denpasar) in Bali, offering more comprehensive data for this province compared with the national MoH surveillance data, which uses a single sentinel site (a Public Health Center in Denpasar) in Bali. Our results were generally comparable across different regencies and types of health facility in Bali in terms of the relative frequencies of influenza types/subtypes observed, lending some support for the representativeness of a single sentinel site Bali for the national MoH surveillance system. Nevertheless, there was significant variation in the overall influenza positive rate among ILI cases, which ranged from 12.6% in Tabanan to 27.5% in Denpasar. The high rate in Denpasar might be due to the higher density and/or mobility of the population (given that Denpasar is a major tourist destination on the island) facilitating increased transmission of influenza viruses to and within this area. However, it is important to acknowledge that differences in influenza positive rates among ILI patients may not reflect differences in influenza attack rates, and could be biased by heterogeneities in other factors, such as in health seeking behavior, between regencies.

The year-round influenza activity we observed in Bali, along with periods of peak activity coinciding with the wet season, is typical of patterns previously observed in tropical regions [26, 27], including Indonesia [24, 28]. Here we found influenza A virus activity to be more strongly associated with the wet season than influenza B, and indeed onset of symptoms during the wet season was the strongest predictor of influenza A infections among ILI patients. In terms of clinical presentation, fever and cough are usually considered the most relevant symptoms of influenza infection [29–31]. While we found that ILI patients with measured fever at presentation with cutoff point ≥38 °C were more likely to have laboratory-confirmed influenza infection, and particularly influenza B, cough was not significantly associated with influenza detection. In fact we found rhinorrhea and chest pain to be more strongly associated with influenza A/H3N2 (but not influenza A/H1N1-pdm09 or influenza B) compared with fever or cough.

Almost 60% of ILI cases and of laboratory-confirmed influenza cases in this study represented the pediatric population (infants, children and adolescents up to 14 years old), consistent with the higher clinical attack rates often observed for influenza in these age groups [18, 25, 32, 33], and indicating children to be more susceptible to, and the main reservoir of, influenza viruses in this setting [34]. Although again, given that ours was not a population-based study design, differences in health seeking behavior could also have a substantial impact on the age-distribution of ILI and influenza cases that we observed. Influenza detection rates among ILI cases tended to be lower in the youngest age group (0–4 years), which could reflect high incidence of other ILI etiologies, such as respiratory syncytial virus, in young children [22].

Previous studies suggest that age profiles for risk of influenza infection can be partly explained by social mixing patterns, with mathematical models that are parameterized using such data better able to capture observed patterns of influenza spread, such as higher attack rates among 5–19 year olds during the initial stages of an epidemic [35, 36]. Here, we found that that influenza positive ILI patients tended to have a higher number of self-reported average daily contacts, although this association was not significant when adjusting for other variables.

Our findings show high rates of ownership of, and physical contact with, both poultry and pigs among ILI cases in Bali. Interestingly, recent physical contact with poultry and pigs were both significantly associated with laboratory confirmed influenza among ILI cases in univariate analysis. It should be emphasized that this result is unlikely to reflect a causal relationship relating to zoonotically acquired infections, given our focus on laboratory detection of human influenza viruses (except for laboratory confirmation of two suspected A/H5N1 cases), and the loss of statistical significance when adjusting for other variables. Nevertheless, these findings do further highlight the potential for cross-species transmission of influenza viruses in this setting, including reverse zoonotic transmission from infected humans to animals which could increase risk of re-assortment between human and animal viruses. Genetic sequencing of viruses isolates is underway to investigate whether any of the infections may represent reassortant or zoonotic strains.

Further molecular studies will also aim to characterize the influenza A positive samples which could not be subtyped using our standard PCR assays. It is not uncommon for influenza A positives of indeterminate subtype to be detected in influenza surveillance, which can result from low viral loads in the nasopharyngeal samples, imperfect sensitivity of the subtyping assays, and genetic drift at the target sites of the PCR primers. Nonetheless, the significantly higher hospitalization rate observed among ILI cases with unsubtyped influenza A (but not those with confirmed A/H1N1-pdm09 or A/H3N2 infection), at over three times the hospitalization rate of influenza-negative cases, is interesting and warrants further investigation.

## Conclusions

In conclusion, over four years of active sentinel surveillance in Bali, Indonesia, between 2010 and 2014, 22% of ILI cases were associated with laboratory confirmed influenza infection, with year-round influenza virus circulation on the island and higher influenza activity during the wet season. High rates of ownership and contact with poultry and pigs were reported among ILI cases, indicating the potential for cross-species influenza transmission in this setting. Genetic characterisation and molecular epidemiological studies are needed to gain insights into the epidemiological and evolutionary dynamics of influenza in Bali, and for early detection of novel reassortant viruses.

## Additional file

Additional file 1: **Table S1.** Univariable analysis of clinical variables for associations with laboratory confirmed influenza among ILI patients. In this supporting information, we present a table showing univariate analysis to assess association between several clinical presentations and laboratory confirmed influenza among ILI patients, including any Influenza (A or B), influenza A/H1N1-pdm09, influenza A/H3N2, and influenza B. (DOCX 23 kb)

## Abbreviations

ILI: Influenza-like illness
MoH: Ministry of Health
RT-PCR: Reserve transcription-Polymerase Chain Reaction
