# Supplementary material for: Surveillance and characterisation of influenza viruses among patients with influenza-like illness in Bali, Indonesia, July 2010–June 2014
Source: BMC Infect Dis. 2019 Mar 7;19:231. doi: 10.1186/s12879-019-3842-5 (PMC6407202; doi:10.1186/s12879-019-3842-5)
Supplement: Supplementary file 1 — Table S1. Univariable analysis of clinical variables for associations with laboratory confirmed influenza among ILI patients. In this supporting information, we present a table showing univariate analysis to assess association between several clinical presentations and laboratory confirmed influenza among ILI patients, including any Influenza (A or B), influenza A/H1N1-pdm09, influenza A/H3N2, and influenza B. (DOCX 23 kb) [file 12879_2019_3842_MOESM1_ESM.docx]

**Additional file**

Table S1. Univariable analysis of clinical variables for associations with laboratory confirmed influenza among ILI patients.

|  | **Any Influenza (A or B)** | | |  | **Influenza A/H1N1-pdm09** | | |  | **Influenza A/H3N2** | | |  | **Influenza B** | | |
| --- | --- | --- | --- | --- | --- | --- | --- | --- | --- | --- | --- | --- | --- | --- | --- |
|  | **OR** | **(95% CI)** | ***P*** |  | **OR** | **(95% CI)** | ***P*** |  | **OR** | **(95% CI)** | ***P*** |  | **OR** | **(95% CI)** | ***P*** |
| Symptom onset in wet season | **2.47** | **(1.96,3.10)** | **<0.01** |  | **3.35** | **(1.90,5.90)** | **<0.01** |  | **5.00** | **(3.29,7.59)** | **<0.01** |  | **1.45** | **(1.04,2.02)** | **0.03** |
| Symptoms: |  |  |  |  |  |  |  |  |  |  |  |  |  |  |  |
| Measured fever ≥38^o^C | **1.41** | **(1.10,1.80)** | **<0.01** |  | 1.50 | (0.86,2.64) | 0.19 |  | 1.20 | (0.85,1.69) | 0.31 |  | **1.54** | **(1.05,2.26)** | **0.02** |
| Measured fever ≥39^o^C | 1.34 | (0.99,1.80) | 0.05 |  | **1.93** | **(1.09,3.42)** | **0.03** |  | 1.42 | (0.95,2.13) | 0.08 |  | 0.65 | (0.38,1.12) | 0.15 |
| Cough | 1.19 | (0.84,1.68) | 0.35 |  | 1.27 | (0.58,2.79) | 0.71 |  | 1.58 | (0.89,2.77) | 0.14 |  | 0.84 | (0.52,1.36) | 0.52 |
| Rhinorrhoea | 1.14 | (0.90,1.43) | 0.3 |  | 0.69 | (0.43,1.09) | 0.13 |  | **1.65** | **(1.15,2.37)** | **<0.01** |  | 0.97 | (0.69,1.37) | 0.86 |
| Headache | 0.99 | (0.80,1.22) | 0.96 |  | 0.92 | (0.59,1.45) | 0.73 |  | 0.92 | (0.68,1.25) | 0.65 |  | 1.22 | (0.89,1.68) | 0.23 |
| Sore throat | 0.87 | (0.70,1.07) | 0.2 |  | 0.73 | (0.46,1.15) | 0.21 |  | 0.85 | (0.63,1.14) | 0.29 |  | 1.00 | (0.73,1.37) | 1 |
| Excess sputum | 0.88 | (0.69,1.14) | 0.37 |  | 1.40 | (0.86,2.32) | 0.17 |  | 0.81 | (0.56,1.19) | 0.32 |  | 0.73 | (0.48,1.10) | 0.15 |
| Vomiting | 0.98 | (0.76,1.26) | 0.9 |  | 1.47 | (0.89,2.42) | 0.13 |  | 0.76 | (0.51,1.12) | 0.19 |  | 1.08 | (0.74,1.57) | 0.7 |
| Diarrhoea | 0.96 | (0.65,1.41) | 0.92 |  | 1.32 | (0.62,2.78) | 0.52 |  | 1.00 | (0.58,1.74) | 1 |  | 0.64 | (0.32,1.28) | 0.23 |
| Chest pain | 1.49 | (0.87,2.54) | 0.14 |  | 0.75 | (0.18,3.11) | 0.59 |  | **2.19** | **(1.15,4.18)** | **0.02** |  | 0.70 | (0.25,1.95) | 0.65 |
| Fatigue | 0.83 | (0.51,1.33) | 0.49 |  | 1.14 | (0.45,2.89) | 0.8 |  | 0.83 | (0.41,1.67) | 0.74 |  | 0.61 | (0.26,1.41) | 0.29 |
| Myalgia | 1.06 | (0.69,1.65) | 0.82 |  | 1.08 | (0.43,2.73) | 0.81 |  | 0.99 | (0.52,1.88) | 1 |  | 0.90 | (0.45,1.81) | 1 |
| Short of breath | 0.74 | (0.45,1.21) | 0.25 |  | 1.15 | (0.46,2.92) | 0.8 |  | 0.54 | (0.23,1.24) | 0.18 |  | 0.73 | (0.33,1.59) | 0.59 |
| Abdominal pain | 0.87 | (0.54,1.40) | 0.64 |  | 1.46 | (0.62,3.44) | 0.32 |  | **0.36** | **(0.13,0.98)** | **0.04** |  | 1.13 | (0.58,2.22) | 0.72 |
| Facility type (ref: hospital) |  |  |  |  |  |  |  |  |  |  |  |  |  |  |  |
| Primary health centre | 0.98 | (0.77,1.25) | 0.90 |  | 0.85 | (0.52,1.41) | 0.51 |  | 1.44 | (0.98,2.09) | 0.06 |  | 0.77 | (0.55,1.10) | 0.16 |
| Treatments prescribed: |  |  |  |  |  |  |  |  |  |  |  |  |  |  |  |
| Antiviral | 1.48 | (0.80,2.72) | 0.23 |  | 0.97 | (0.23,4.08) | 1 |  | **2.34** | **(1.15,4.75)** | **0.03** |  | 1.33 | (0.52,3.41) | 0.59 |
| Oseltamivir | 1.44 | (0.32,6.37) | 0.73 |  | 0.33 | (0.02,5.79) | 0.46 |  | **1.42** | **(1.14,1.76)** | **0.04** |  | 0.31 | (0.04,2.51) | 0.28 |
| Antibiotics | 0.95 | (0.73,1.23) | 0.68 |  | 1.48 | (0.79,2.77) | 0.25 |  | 0.88 | (0.61,1.28) | 0.5 |  | 0.86 | (0.57,1.29) | 0.46 |
| Time to seek treatment (per day increase) | 0.99 | (0.99,1.00) | 0.14 |  | 1.00 | (0.99,1.00) | 0.63 |  | 0.98 | (0.94,1.01) | 0.17 |  | 0.99 | (0.98,1.00) | 0.35 |
